# Validation and Application of an HPLC-UV Method for Routine Therapeutic Drug Monitoring of Dalbavancin

**DOI:** 10.3390/antibiotics11050541

**Published:** 2022-04-19

**Authors:** Ute Chiriac, Heike Rau, Otto R. Frey, Anka C. Röhr, Sabrina Klein, Anna L. Meyer, Benedict Morath

**Affiliations:** 1 Department of Pharmacy, Heidelberg University Hospital, 69120 Heidelberg, Germany; benedict.morath@med.uni-heidelberg.de; 2 Department of Pharmacy, General Hospital of Heidenheim, 89522 Heidenheim, Germany; heike.rau@kliniken-heidenheim.de (H.R.); otto.frey@kliniken-heidenheim.de (O.R.F.); anka.roehr@kliniken-heidenheim.de (A.C.R.); 3 Department of Infectious Diseases, Medical Microbiology and Hospital Hygiene, Heidelberg University Hospital, 69120 Heidelberg, Germany; sabrina.klein@med.uni-heidelberg.de; 4 Department of Cardiac Surgery, Heidelberg University Hospital, 69120 Heidelberg, Germany; anna.meyer@med.uni-heidelberg.de; 5 Cooperation Unit Clinical Pharmacy, Heidelberg University, 69120 Heidelberg, Germany

**Keywords:** dalbavancin, pharmacokinetic/pharmacodynamics (PK/PD), therapeutic drug monitoring (TDM), high performance liquid chromatography–ultraviolet spectrometry (HPLC–UV)

## Abstract

Dalbavancin is emerging as a promising alternative in the ambulant treatment of gram-positive infections that require long-term antibiotic treatment such as osteomyelitis, prosthetic joint infections, and endocarditis. The aim of the current study was to develop and validate a simple, rapid, and cost-effective high-performance liquid chromatography–ultraviolet spectrometry (HPLC–UV) method for the quantification of dalbavancin. Sample clean-up included a protein precipitation protocol, followed by chromatographic separation on a reverse phase HPLC column (C-18) with gradient elution of the mobile phase. Quantification was performed with the internal standard (caffeine) method. Linear relationships between peak area responses and drug concentrations were obtained in the range of 12.5–400 mg/L. The variation coefficient of precision and the bias of accuracy (both inter- and intraday) were less than 10%. The limit of quantification (LOQ) was 12.5 mg/L. The simple and reliable HPLC–UV assay described is a powerful tool for routine therapeutic drug monitoring (TDM) of dalbavancin in human serum in clinical laboratories. With a total process time of approximately 20 min, it allows for accurate and selective quantification up to the expected pharmacokinetic peak concentrations. The method was successfully used to analyze subsequent serum samples of three patients and showed good performance in monitoring serum levels.

## 1. Introduction

Dalbavancin is a novel, long-acting glycopeptide indicated for the treatment of acute bacterial skin and skin structure infections (ABSSSIs) caused by Gram-positive bacteria, including multi-drug-resistant strains [1,2]. However, an ever-growing body of evidence supports the efficacy of dalbavancin as a long-term therapy for off-label indications, in which treatment for at least 6 weeks is usually required, such as osteomyelitis, joint infections, endocarditis, and bacteremia due to an intravascular source [3].

Dalbavancin is promising in relation to both its strong bactericidal activity against different resistance patterns and its unique pharmacokinetic properties; a long elimination half-life (approximately >14 days), high level of protein binding (93%), predominantly non-renal clearance (0.05 L/h), and good tissue penetration represent its main pharmacokinetic properties [4]. Efficacy is associated with the area under the concentration-time curve (AUC) divided by the minimal inhibitory concentration (MIC). Experimental data suggest a free AUC/MIC_0–24_ value of 27.1 for bacterial stasis, 53.3 for 1-log kill, and 111.1 for 2-log kill against *Staphylococcus aureus* [5]. The epidemiological cut-off value (ECOFF) of *Staphylococcus aureus* is currently set at 0.25 mg/L by the European Society of Clinical Microbiology and Infectious Diseases (EUCAST) [6]. Thus, the unique pharmacokinetic (PK)/pharmacodynamic (PD) properties of dalbavancin make it a valuable alternative for daily in-hospital intravenous regimens in the treatment of infections requiring long-term antibiotic therapy.

Clinical evidence for dalbavancin use in long-term treatment shows a wide heterogeneity in dalbavancin dosing schedules and treatment duration across different clinical scenarios. Dunne et al. [7] suggest that only two doses of dalbavancin (1500 mg on day 1 and 1500 mg on day 8) may provide sufficient tissue exposure in *Staphylococcus aureus* infections for 8 weeks. Therefore, routine therapeutic drug monitoring (TDM) of dalbavancin would contribute to the efficacy and safety of the novel, long-acting glycopeptide for use in in long-term treatments. To date, there is only one high-performance liquid chromatography (HPLC) method with mass spectrometry (MS) for the quantification of dalbavancin that has been reported [8]. Still, the implementation and use of HPLC–MS/MS techniques usually requires well-trained staff, specialized laboratories, and institutes that can afford these cost- and maintenance-intensive devices. Additionally, chemical reagents and special reference substances are needed for HPLC–MS/MS analytics, resulting in higher costs per measurement. In comparison, HPLC methods with ultraviolet (UV) detection usually have rather long run times, lower detection capabilities, and lower selectivity than HPLC–MS/MS. However, HPLC–UV is widely represented in smaller laboratories and is already applied for routine analytical purposes in hospital pharmacies.

Therefore, we aimed to develop a simple, rapid, and cost-effective HPLC–UV method for the quantification of dalbavancin doses suitable for routine TDM.

## 2. Results

**Figure 1:**
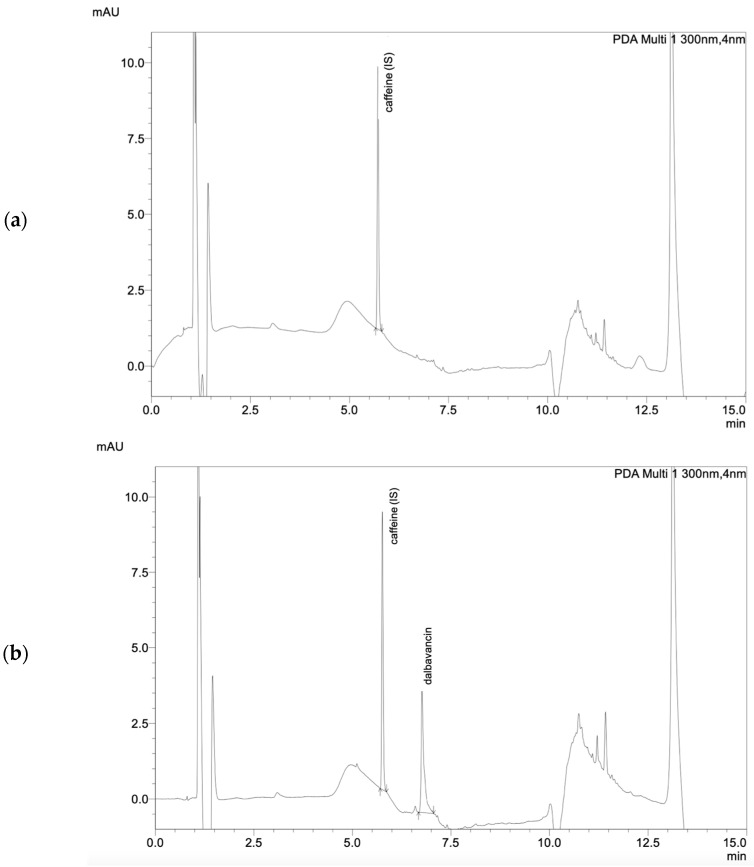

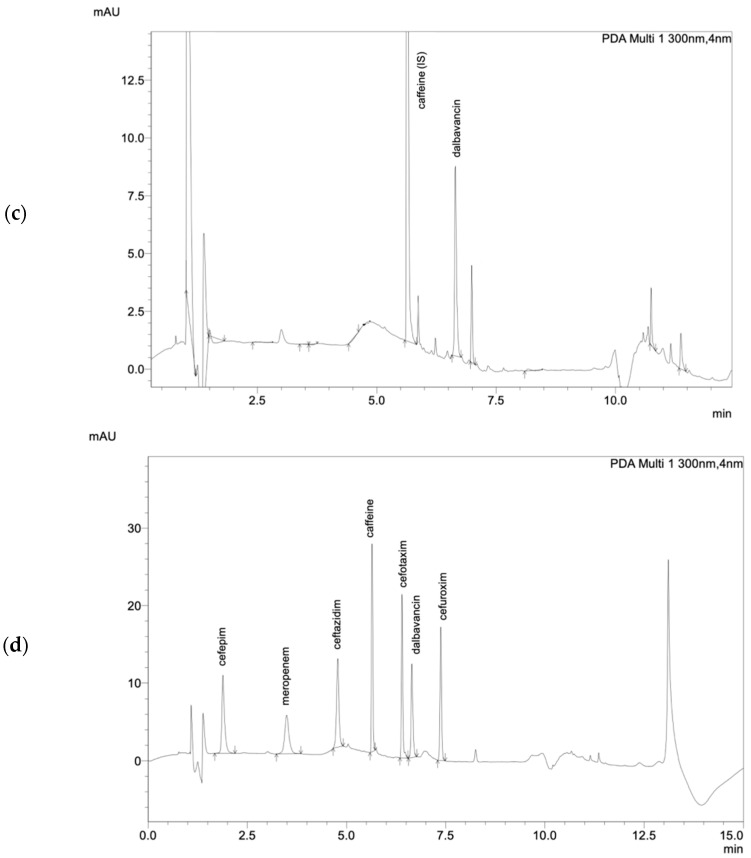
(**a**) Chromatogram of blank serum, (**b**) chromatogram of dalbavancin calibration standard (200 mg/L), (**c**) chromatogram of dalbavancin in patient serum and (**d**) chromatogram showing cefepime, meropenem, ceftazidime, caffeine, cefotaxime, dalbavancin, and cefuroxime.

**Figure 2:**
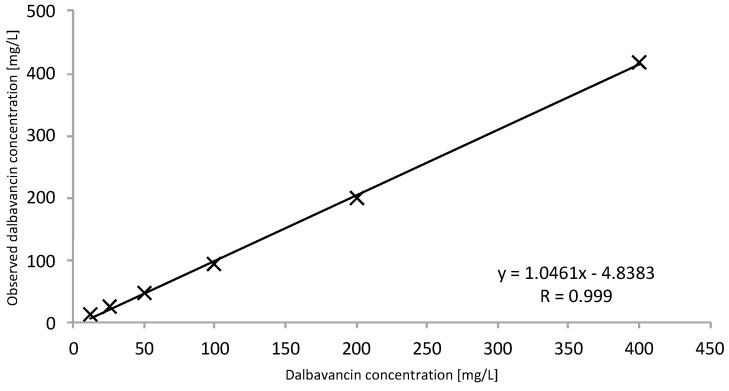
Calibration curve to determine total dalbavancin. A calibration curve was performed with serum calibration standards at 12.5, 25, 50, 100, 200, and 400 mg/L concentrations (R = 0.999).

The tested and validated HPLC–UV method allows for the easy detection of total dalbavancin in human serum (Figure 1). Sufficient peak separation from other antibiotics quantifiable with this method was achieved with the given HPLC–UV parameters. Dalbavancin peaks could be identified at a retention time of 6.65 min at a wavelength of 300 nm. The method was in line with the Valistat 2.0 (ARVECON GmbH, Walldorf, Germany) validation criteria as required by the German Society of Toxicology and Forensic Chemistry (GTFCh) [9]. The calibration curve was linear over the concentration range of 12.5 to 400 mg/L, with a correlation coefficient of >0.99 (Figure 2). The bias for accuracy within the samples analyzed on different days ranged between −2 to 0%. The lower limit of quantification (LOQ) was determined to be 12.5 mg/L. The limit of detection (LOD) was determined to be 6.25 mg/L. All acceptance criteria were applied and fulfilled for precision and for inter- and intraday assay performance. Inter- and intraday precision showed a low overall variation coefficient of 0.2 to 4%, indicating good assay performance. Mean recovery values were greater than 76%. There was no evidence of interference of other drugs at the given retention time and wavelength. Detailed validation data are presented in the Supplementary Materials (Tables S1–S3).

The stability of dalbavancin was very high, as described in other studies [10]. At room temperature, samples were stable at least up to 48 h. Stability continued for at least 96 h when refrigerated (2–8 °C), at least 1 month when frozen at −20 °C, and at least 12 months when frozen at −80 °C. The stability of the aqueous stock solution was stable for at least 3 months when refrigerated (2–8 °C) and at least 12 months when frozen at −80 °C (Table S4).

The method was successfully used to analyze serum samples of three cardiologic patients receiving dalbavancin for infection with *Staphylococcus aureus*. Serum samples were obtained as part of routine care, which includes the determination of dalbavancin concentrations as part of a TDM service, and patients consented to the publication of the results. The samples were subsequently analyzed according to the described method. The measured dalbavancin concentrations in serum are described in Table 1. No interference with other prescribed drugs was observed in these patients. Moreover, peak purity was monitored and continuously present when analyzing dalbavancin in the serum samples of patients. The described patients were treated with dalbavancin for wound infection due to *Staphylococcus aureus*. The serum dalbavancin concentrations were obtained 1 h after the first infusion (peak level, c_max_) on day one of therapy and right before the next infusion on day 8 (trough level, c_min_). The patients’ estimated glomerular filtration (eGFR) was calculated by the chronic kidney disease epidemiology collaboration equation (CKD–EPI) [11]. The AUC was calculated using the trapezoidal rule.

## 3. Discussion

We report a simple and cost-effective method for the quantification of total dalbavancin in human serum for daily routine use with short turnaround times. The provided method had high accuracy and selectivity for clinically observed drug concentrations, as clinical studies reported concentrations ranging between 18 and 411 mg/L [2,10,13]. Moreover, the LOQ of 12.5 mg/L was below the reported concentrations, but still allowed for the sufficient measurement of concentrations above the required ECOFF of 0.25 mg/L for *Staphylococcus aureus*. Compared to a previously reported method with MS/MS detection, this method showed a higher LOQ [8]. However, quantification on the forensic scale is not necessary, as TDM is the major application of this method. In laboratories that frequently perform TDM of beta-lactam antibiotics, an integration of dalbavancin in existing HPLC–UV methods with short turnaround times is a reasonable way to minimize cost and effort.

The chosen internal standard caffeine was a valid and easily accessible substance to monitor assay performance. Since caffeine is rarely used for drug therapies, misinterpretation of chromatograms by false high internal standard peaks is negligible. As the reported HPLC–UV method is already implemented for quantifying beta-lactam antibiotics (e.g., meropenem, piperacillin), multiple chromatograms of the TDM performed were available [14]. No interferences were shown at the given retention time and wavelength of dalbavancin. Thus, this method allows for the determination of dalbavancin in the presence of co-administered vancomycin, flucloxacillin, or cephazoline. These drugs are usually the first choice prescribed for patients with gram-positive infections and with an indication for dalbavancin treatment. Therefore, accuracy and precision were determined with these compounds.. As significant amounts of metabolites have not been detected in human serum, further evaluation of metabolites was dispensed [15]. Stability studies showed high dalbavancin stability in spiked human serum. If sample processing is delayed for more than 96 h, storage conditions should be set to −20 °C, or if for more than 1 month, to −80 °C.

Determination of dalbavancin in serum poses many challenges due to dalbavancin’s protein binding and high adsorption properties in collection containers or materials [8]. This might explain the different recovery rate values observed in other matrices (pooled serum of critically ill patients, sheep serum in Table S3). Every minor change in the materials and methods could require a review of the recovery rate due to these properties. In particular, the determination of the free fraction is limited by the strong adsorption of dalbavancin to most of commonly used materials. Standard in vitro methods for determining the free fraction, such as ultrafiltration, were unfeasible. Microdialysis was not an option in our case as it is an invasive procedure. For this reason, we developed a sample preparation method with a solid phase extraction using Nanosept^®^. However, this method is very complex and time-consuming, and thus is not very suitable for routine monitoring in clinical practice. In contrast to clinical approval studies describing an extensive protein binding of 93% [4,12], we observed a higher protein binding of between 96 and 98% in three samples of cardiologic patients. Additionally, we observed protein binding ranging between 92 and 98% in spiked human serum (Table S5). Dalbavancin total concentrations below the LOQ of 12.5 mg/L would be expected to have a maximum free fraction of 0.9 mg/L and an *f*AUC of 21.0 mg∙L/h according to the literature, while we observed a maximum free fraction of 0.4 mg/L and an *f*AUC of 9.0 mg∙L/h, in accordance with the higher protein binding. Consequently, patients with long-term antibiotic therapy might benefit from a redosing if dalbavancin total concentrations fall below 12.5 mg/L to cover the ECOFF of 0.25 mg/L for *Staphylococcus aureus*. To our knowledge, a reference method for determining the free fraction of dalbavancin in serum is not yet available. Therefore, an independent method should be developed to validate this observation.

The measured total dalbavancin concentrations from the three cardiologic patients were well within the concentrations of the calibration range. The observed peak concentrations after the initial dosing were 201–278 mg/L. Trough concentrations on day 8 before redosing were 23.0–44.9 mg/L, corresponding to a *f*AUC of 38.4–74.4 mg∙L/h, which is sufficient according to the literature. These findings again demonstrate the existence and moreover the extent of variability in pharmacokinetics, which is not limited to critically ill patients but also applies to multimorbid patients and is of major importance for optimizing antibiotic therapy. Due to the novelty and antimicrobial activity of dalbavancin, its use is currently exceptional, and only three patients were eligible for TDM. Long-term antibiotic therapy with dalbavancin is a desirable option for managing patients with severe or persistent infection in an outpatient setting. The current COVID-19 pandemic, in which unnecessary hospitalizations should be avoided and ambulant therapy empowered, further strengthens the role of feasible outpatient antimicrobial management strategies [3]. Oral substances such as fluoroquinolones, clindamycin, linezolid, or cotrimoxazole that are currently used for long-term treatment are often limited by the occurrence of adverse events, drug interactions, or complications such as an increased risk of *Clostridioides difficile* infections [16,17]. In addition, these options further increase the pill burden in patients who are often already treated with multiple drugs [18,19]. This might negatively affect adherence, leading to therapy failure—not only of the antibiotic regimen but also of the overall medication regime [20,21]. Outpatient parenteral therapy is a mainstay for these patients in the US but is not widely available in some other countries (e.g., Germany) [22,23]. Nevertheless, outpatient parenteral therapy can be associated with complications and close monitoring is recommended [22].

The current method provides the opportunity to monitor dalbavancin concentrations, thereby ensuring its safety and efficacy during the use of dalbavancin for long-term treatment in off-label indications or vulnerable patient populations. TDM might not be indicated in all patients, but it can be of particular importance in special patient populations such as obese patients or patients with organ dysfunction to prevent under- and overdosing [24]. In particular, in patients treated with dalbavancin as suppression therapy for persistent infection, PK/PD target attainment is crucial to prevent exacerbation of underlying infections.

## 4. Material and Methods

### 4.1. Calibrators Samples, Quality Control Samples and Internal Standard

All used solvents were of HPLC or comparable quality and all reagents were of analytical grade. Dalbavancin was purchased from Allergan Pharmaceutial International (Xydalba^®^, Dublin, Ireland) as a regular vial containing powder for reconstitution. Human serum was purchased from Sigma-Aldrich (Steinheim, Germany). Ultrapure water was obtained from Fresenius Kabi (Ampuwa^®^, Frankfurt Rhein-Main, Germany). Caffeine was obtained from Merck (Darmstadt, Germany).

Stock solution of dalbavancin was prepared by dissolving 500 mg dalbavancin powder in 250 mL of ultrapure water to get a 2000 mg/L. The internal standard was dissolved in acetonitrile/methanol (1:1) mixture at a concentration of 50 mg/L.

Serum calibration standards with 12.5, 25, 50, 100, 200 and 400 mg/L concentrations of dalbavancin, and serum quality control concentrations of 50 (low concentration, LQC), 100 (medium concentration, MQC) and 200 (high concentration, HQC) mg/L were prepared by adding appropriate volumes of dalbavancin stock solution to human serum. Aqueous calibration standards with 1, 2.5, 5, 7.5 and 10 mg/L concentrations of dalbavancin were prepared by adding appropriate volumes of dalbavancin stock solutions to ultrapure water. All solutions were stored as 500 µL aliquots in polypropylene Eppendorf tubes at −80 °C and thawed just before use.

### 4.2. Sample Preparation

To determine total dalbavancin in serum, protein precipitation was performed by adding 200 µL of an acetonitrile/methanol (1:1) mixture with 50 mg/L caffeine to 100 µL patient serum. Subsequently, samples were mixed for 10 s, stored in an ultrasonic bath for 10 min at 40 °C, then centrifuged at 8000× *g* for 5 min. Next, 100 µL of the resulting supernatant was further diluted with 500 µL water with 0.1% formic acid. In addition, the free fraction of dalbavancin was determined by solid phase extraction using a centrifugal device (Nanosep^®^, Pall, New York, NY, USA). Details are described in the Supplementary Materials (Figures S1–S3).

### 4.3. HPLC Conditions

An aliquot of 20 µL was injected onto the HPLC–UV system equipped with a diode array detector (Nexera-I 3D plus, Shimadzu, Duisburg, Germany). Chromatographic analysis was performed using a reversed phase column Shim-pack XR-ODS III with 2.2 µm particle size (150 mm × 2 mm, Shimadzu, Duisburg, Germany) in combination with a column guard (Shim-pack GISS-H (G) C18, 3 µm; 10 mm × 2.1 mm, Shimadzu, Duisburg, Germany). Separation was performed using a gradient of 0.1% formic acid in acetonitrile and 0.1% formic acid in water with a flow rate of 0.35 mL/min. The autosampler and column were set at temperatures of 10 °C and 45 °C, respectively. Dalbavancin was monitored at a wavelength of 300 nm with a retention time of 6.65 min.

### 4.4. Assay Validation

For peak identification, aqueous solutions of dalbavancin (200 mg/L) were analyzed with the above-mentioned method and assessed for intensity (area), shape, and retention time. Further assay validation was performed with spiked human serum.

The linearity of total dalbavancin was conducted via a calibration curve of serum calibration standards. Each of the serum calibration standards was analyzed six times and evaluated by peak area as well as peak height vs. target concentration, with an acceptable correlation coefficient of >0.95. The LOQ was set at the lowest standard calibration concentration (12.5 mg/L); this also corresponds to a signal-to-noise ratio of 6:1. The LOD was estimated at a signal-to-noise ratio of 3:1.

In order to determine assay precision, the coefficient of variation (CV, reported in %) of serum quality control concentrations was calculated. Differentiation between inter- and intraday precision was performed in addition, with intraday precision (repeatability) being determined three times during the same day, while interday precision (reproducibility) being determined once a day on eight consecutive days. A general coefficient of variation of ±15% was accepted. Accuracy was evaluated by using the same samples as described, for precision, at three different concentrations on eight different days. The degree of accuracy was determined by the bias, with ± 15% being acceptable. The recovery was determined at a LQC and a HQC run in six replicates each. The absolute recoveries (%) were calculated by comparing the peak areas of dalbavancin in spiked human serum, extracted as described, with those of spiked aqueous solutions at the same concentration levels not subjected to the extraction procedure.

Furthermore, interference with other drugs was assessed by cross-examination of 50 chromatograms of patients not receiving dalbavancin, but other anti-infectives and other intensive care specific drugs, as well as six chromatograms of different blank sera. The chromatograms were examined for interfering peaks at the retention time and detection wavelength of 300 nm for dalbavancin. Additionally, a complete UV spectrum was checked for peak purity and identification.

### 4.5. Stability

The stability of dalbavancin in human serum, and water for injection, was studied at different conditions including room temperature, refrigeration at 2–8 °C, and freezing at −20 °C as well as −80 °C. For stability testing, two samples were thawed out and subsequently analyzed. Stability was defined by the time for which samples remained a concentration of > 90% of their baseline concentrations.

## 5. Conclusions

Dalbavancin is an attractive option for outpatient antibiotic therapy, including patients requiring long-term therapy. Since dosing strategy is still a matter of debate and may depend on several factors, TDM may possibly be helpful for monitoring dalbavancin concentrations and preventing long-term drug accumulation or insufficient concentrations. With the described method, dalbavancin TDM can be performed on a daily basis due to a quick turnaround time and can be easily implemented in smaller laboratories or hospital pharmacies due to the cost-effectiveness of HPLC–UV methods. Therefore, this method could contribute to the safe and effective prescribing of dalbavancin in outpatient settings and long-term therapies.

## Figures and Tables

**Table 1:** Dalbavancin concentrations of three cardiologic patients.

Patient	Weight [kg]	Height [cm]	Age [Years]	eGFR [mL/min]	Albumin [mg/L]	Infection	Pathogen	Dose [mg]	c_max(d1)_ [mg/L]	*f*c_max(d1)_ [mg/L] *	*f*c_max(d1)_ [mg/L] **	c_min(d8)_ [mg/L]	*fc_min_*_(d8)_ [mg/L] *	*fc_min_*_(d8)_ [mg/L] **	*f*AUC_(d8)_ [mg∙h/L] *	*f*AUC_(d8)_ [mg∙h/L] **
1	85	175	60	d1: 98 d8: 96	d1: 46.0 d8: n.a.	LVAD infection	*S. aureus*	1500	278	5.4	19.5	44.9	1.3	3.1	31.2	74.4
2	95	174	58	d1: 76 d8: 65	d1: 44.6 d8: 46.3	LVAD infection	*S. aureus*	1500	232	6.4	16.2	36.8	1.0	2.6	24.0	62.4
3	72	176	68	d1: 73 d8: 94	d1: 44.3 d8: 44.7	LVAD infection	*S. aureus*	1500	201	6.7	14.1	23.0	<1.0	1.6	<24.0	38.4

c_max_ peak concentration, c_min_ trough concentration, d day, eGFR estimated glomular filtration, *f*c free concentration, LVAD left ventricular assist device, n.a. not available. * Free concentration determined by solid phase extraction using a centrifugal device (Nanosep^®^, Pall, New York, NY, USA), see also Supplementary Materials Figure S1. ** Free concentration calculated according to approval studies (protein binding of 93%) [4,12].

## Data Availability

No new data were created or analyzed in this study. Data sharing is not applicable to this article.

## Supplementary Materials

The following supporting information can be downloaded at: https://www.mdpi.com/article/10.3390/antibiotics11050541/s1, Figure S1: Determination of the free fraction of dalbavancin, Figure S2: Chromatogram of aqueous dalbavancin calibration standard at 10 mg/L [black], 5 mg/L [green] and 2.5 mg/L [blue] concentration (a) and chromatogram of free dalbavancin in patient serum (b). Dalbavancin was monitored at a wavelength of 300 nm with a retention time of 6.65 min, Figure S3: Calibration curve to determine free dalbavancin with 150 μL aqueous calibration standards at 1, 2.5, 5, 7.5 and 10 mg/L concentration (R = 0.998). The correlation coefficient should be monitored and be above 0.99, Table S1: Calibration values of total dalbavancin in human serum, Table S2: Results of intraday and interday precision (CV%) and accuracy (bias%) of total dalbavancin in human serum, Table S3: Recovery rate of total dalbavancin in human serum, in patient serum (critically ill patient) and in sheep serum, Table S4: Stability of dalbavancin in human serum, Table S5: Dalbavancin total and free concentrations in spiked human serum samples. In order to evaluate the performance spiked human serum samples with a low (50 mg/L) and a high (200 mg/L) dalbavancin concentration were analyzed.
