# Supplementary material for: Validation and Application of an HPLC-UV Method for Routine Therapeutic Drug Monitoring of Dalbavancin
Source: Antibiotics (Basel). 2022 Apr 19;11(5):541. doi: 10.3390/antibiotics11050541 (PMC9137512; doi:10.3390/antibiotics11050541)
Supplement: Supplementary file 1 [file antibiotics-11-00541-s001.zip › antibiotics-1632956-supplementary.pdf]

Figure S1. Determination of the free fraction of dalbavancin.

To determine the free fraction of dalbavancin samples were prepared by solid phase extraction using a centrifugal device (Nanosep®, Pall). The lower limit of quantification (LOQ) was determined to be 1 mg/L.

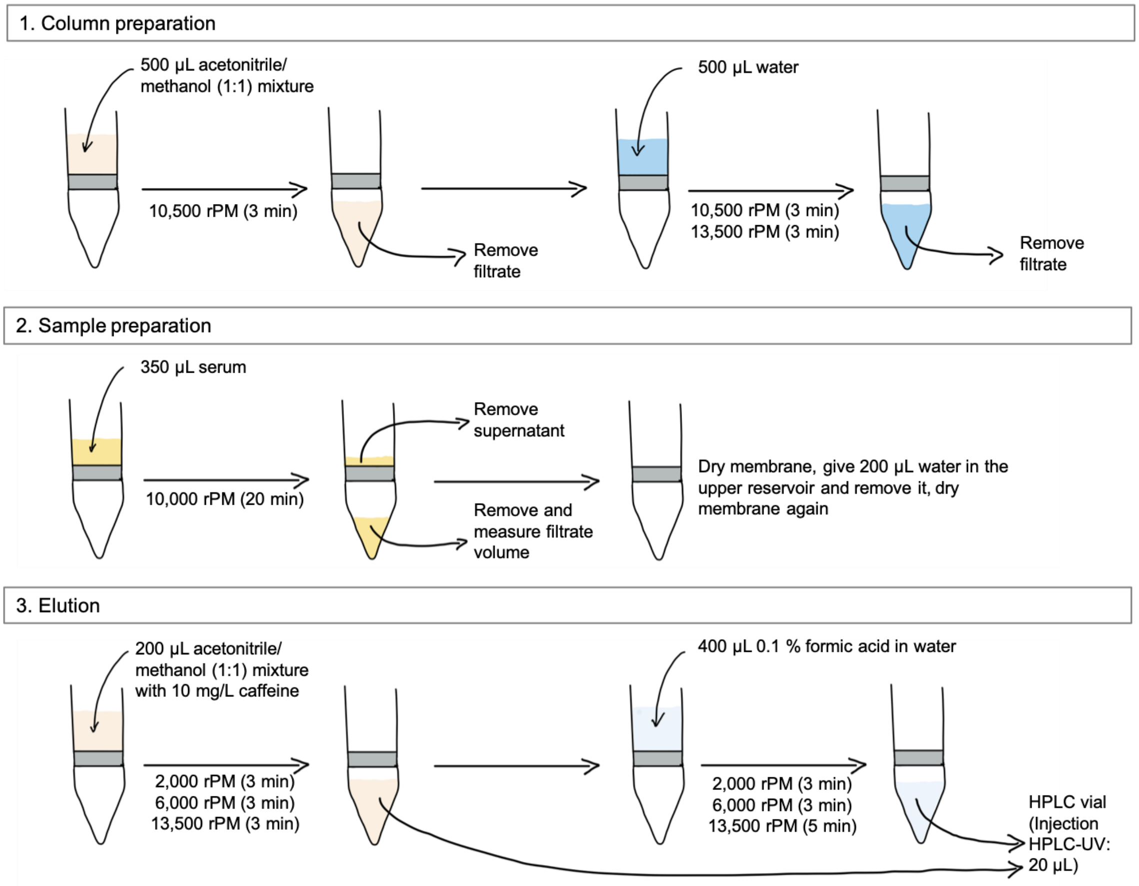

Figure S2. Chromatogram of aqueous dalbavancin calibration standard at 10 mg/L [black], 5 mg/L [green] and 2.5 mg/L [blue] concentration (**a**) and chromatogram of free dalbavancin in patient serum (**b**). Dalbavancin was monitored at a wavelength of 300 nm with a retention time of 6.65 min.

(a)

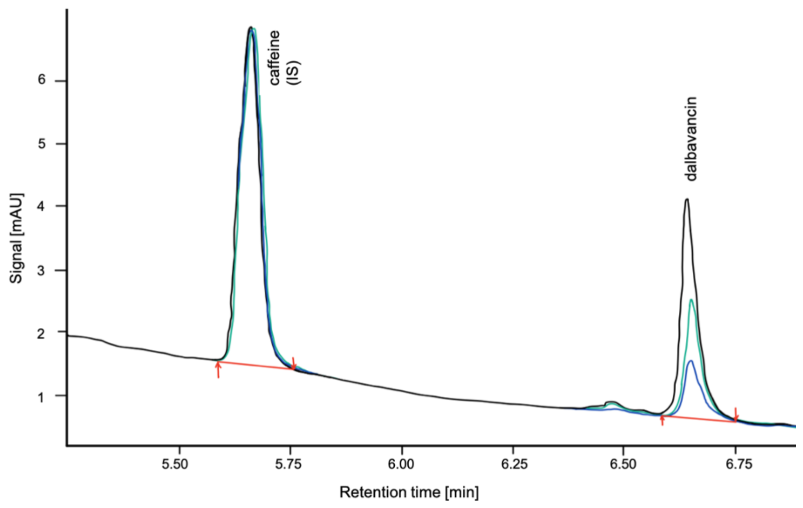

(b)

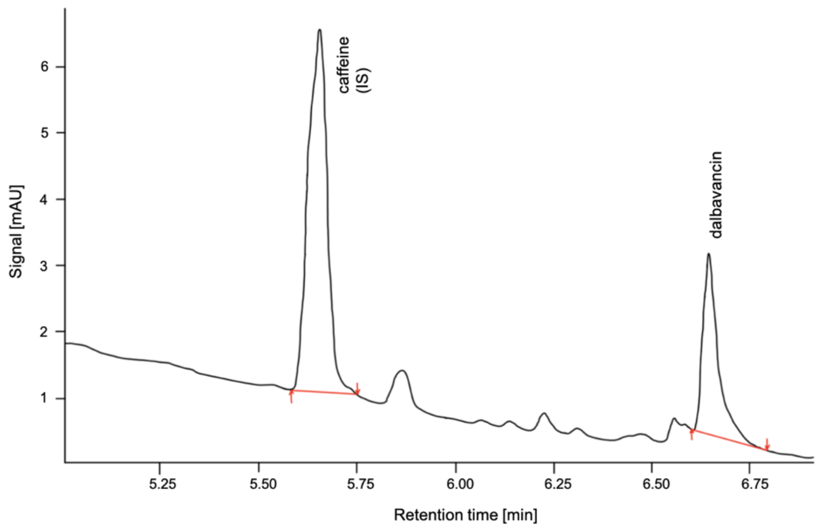

Figure S3. Calibration curve to determine free dalbavancin with 150 µL aqueous calibration standards at 1, 2.5, 5, 7.5 and 10 mg/L concentration (R = 0.998). The correlation coefficient should be monitored and be above 0.99.

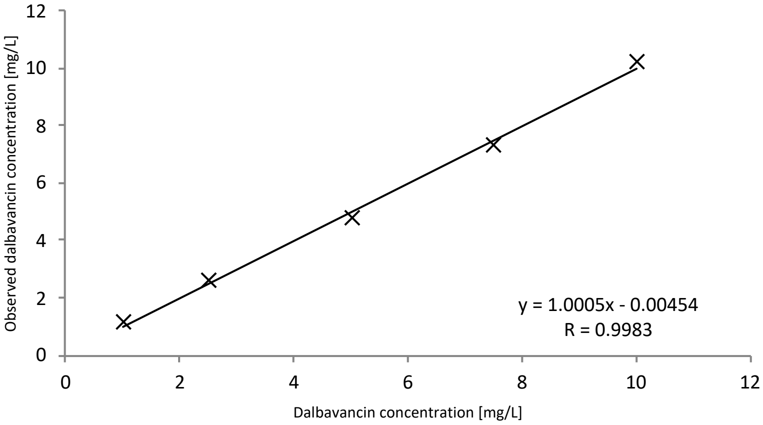

Table S1. Calibration values of total dalbavancin in human serum.

Theoretical concentration [mg/L]	Mean concentration [mg/L]	SD
12.5	12.1	1.46
25	24.5	1.22
50	48.7	1.37
100	92.3	2.07
200	200.0	2.83
400	417.2	4.12

Table S2. Results of intraday and interday precision (CV%) and accuracy (bias%) of total dalbavancin in human serum.

Theoretical concentration [mg/L]	Mean concentration [mg/L]	Intraday [%]	Interday [%]	Bias [%]
50	49.0	3.68	0.26	-2.00
100	98.1	3.06	2.00	-1.91
200	198.1	3.29	0.74	-0.96

Table S3. Recovery rate of total dalbavancin in human serum, in patient serum (critically ill patient) and in sheep serum.

Theoretical concentration [mg/L]	Recovery rate [%] (human serum)	CV [%] (human serum)	Recovery rate [%] (patient serum)	CV [%] (patient serum)	Recovery rate [%] (sheep serum)	CV [%] (sheep serum)
50	77.1	3.6	93.0	2.0	93.5	3.4
200	75.8	1.9	90.8	10.4	92.1	5.2

Table S4. Stability of dalbavancin in human serum.

Theoretical concentration [mg/L]	25 °C 48 h	2-8 °C 96 h	-20 °C 1 month	-80 °C 12 months
222	104 %	103 %	101 %	104 %
58	103 %	98 %	95 %	95 %

Table S5. Dalbavancin total and free concentrations in spiked human serum samples. In order to evaluate the performance spiked human serum samples with a low (50 mg/L) and a high (200 mg/L) dalbavancin concentration were analyzed.

(a) Spiked human serum samples of 14 patients (8 critically ill and 6 non-critically ill) after calibration with sheep serum. Protein binding ranging between 95 and 98 %.

		50 mg/L			200 mg/L		
Patient		c [mg/L]	*f*c [mg/L]	PB [%]	c [mg/L]	*f*c [mg/L]	PB [%]
1	critically ill	53.5	1.4	97	201	7.6	96
2	critically ill	53.6	1.7	97	207	9.0	96
3	critically ill	51.2	1.9	96	209	9.1	96
4	critically ill	54.2	1.6	97	212	7.7	96
5	critically ill	53.0	2.1	96	203	9.5	95
6	critically ill	50.4	1.5	97	204	9.2	95
7	critically ill	49.1	2.4	95	195	10.2	95
8	critically ill	54.3	1.8	97	201	8.7	96
9	non-critically ill	40.0	<1.0	-	177	3.7	98
10	non-critically ill	47.8	<1.0	-	180	4.0	98
11	non-critically ill	38.6	<1.0	-	177	3.5	98
12	non-critically ill	42.6	<1.0	-	195	3.9	98
13	non-critically ill	53.0	1.1	98	214	6.3	97
14	non-critically ill	50.0	1.1	98	208	4.6	98

c concentration, *f*c free concentration, PB protein binding

(b) Spiked human serum samples of 9 critically ill patients and 6 healthy volunteers after calibration with human serum. Protein binding ranging between 92 and 98 %.

		50 mg/L			200 mg/L		
Patient		c [mg/L]	fc [mg/L]	PB [%]	c [mg/L]	fc [mg/L]	PB [%]
15	critically ill	56.7	1.9	97	239	9.8	95
16	critically ill	56.7	1.8	96	233	12.1	94
17	critically ill	52.5	<1.0	-	222	8.3	96
18	critically ill	57.6	1.3	97	242	8.8	96
19	critically ill	51.1	1.2	98	197	7.1	96
20	critically ill	54.5	1.7	97	254	8.2	96
21	critically ill	55.2	1.6	97	230	11.0	94
22	critically ill	59.8	2.5	95	266	16.5	92
23	critically ill	56.0	2.2	96	233	9.6	95
24	healthy volunteer	46.1	1.6	97	184	4.6	98
25	healthy volunteer	45.8	1.6	97	197	5.8	97
26	healthy volunteer	52.6	1.6	97	219	5.2	97
27	healthy volunteer	52.5	1.7	97	220	5.1	97
28	healthy volunteer	49.1	1.6	97	199	4.8	98
29	healthy volunteer	52.0	1.4	97	207	4.6	98

c concentration, *f*c free concentration, PB protein binding
